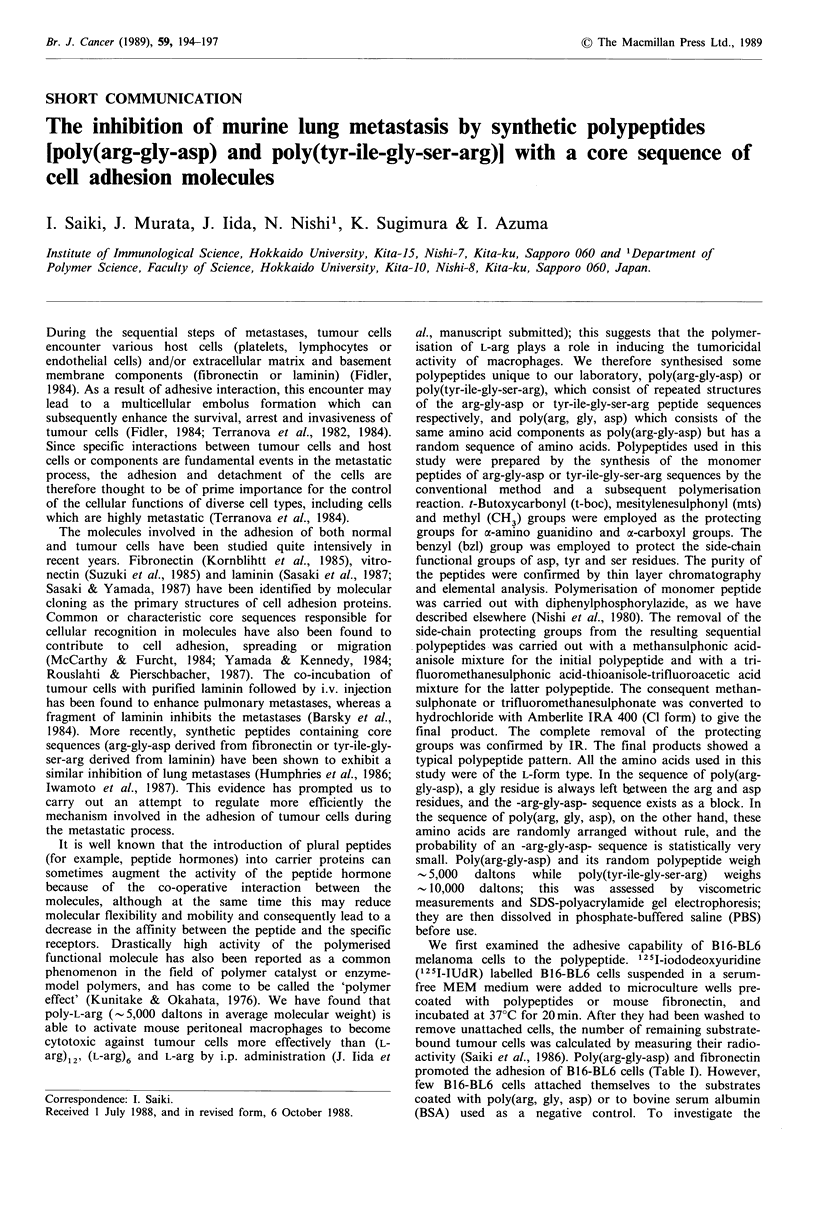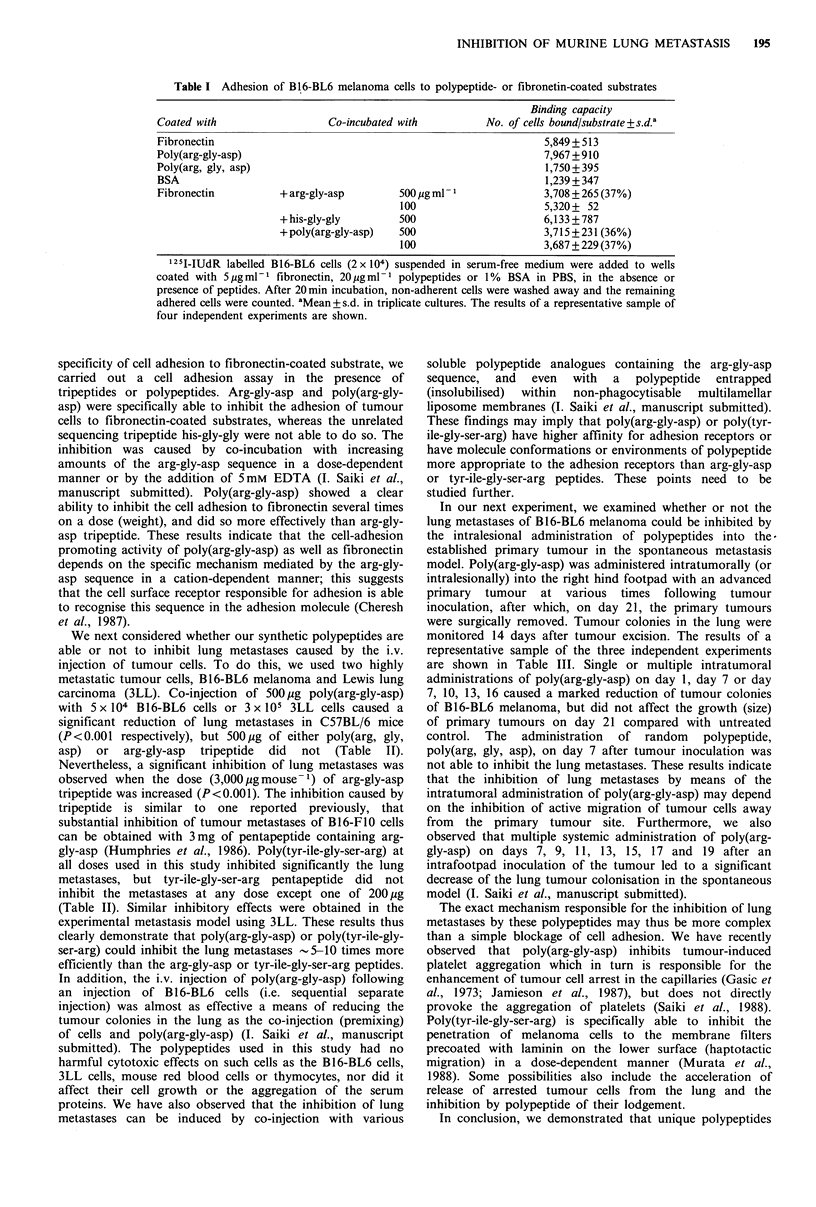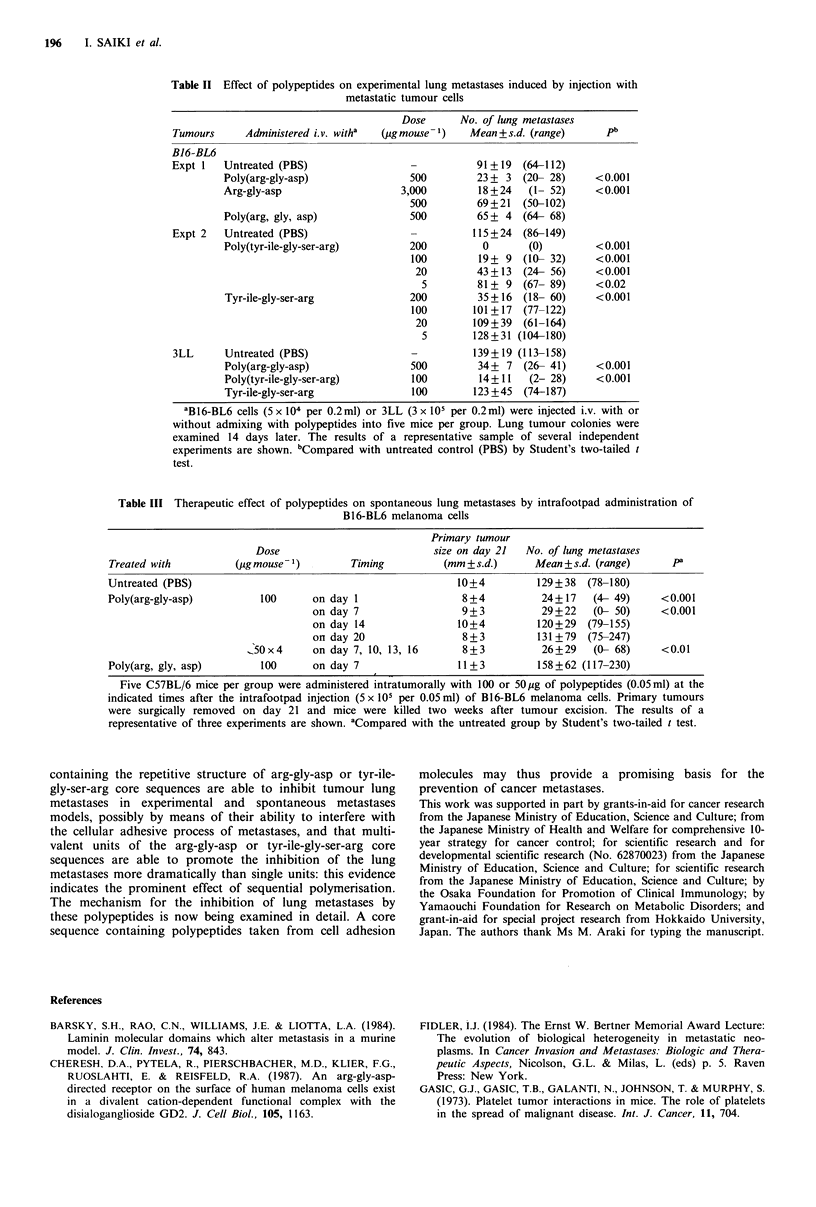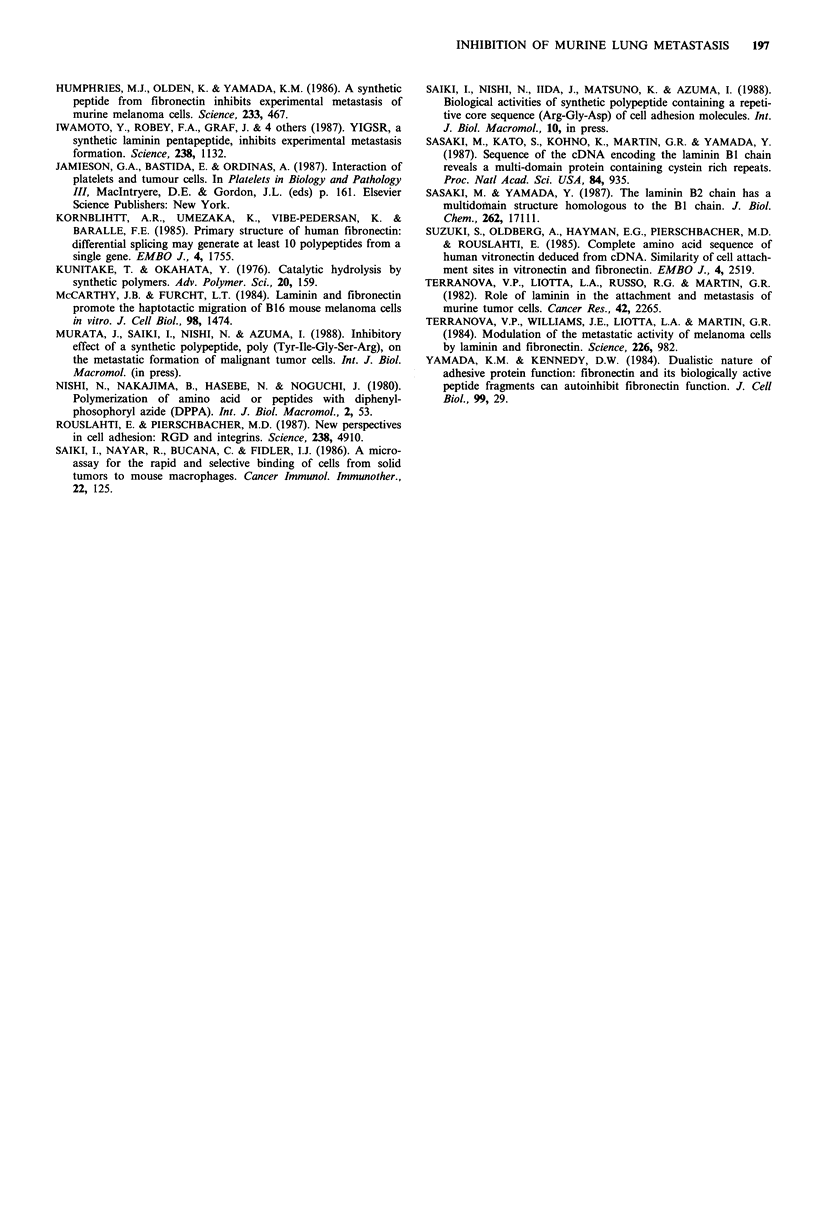# The inhibition of murine lung metastasis by synthetic polypeptides [poly(arg-gly-asp) and poly(tyr-ile-gly-ser-arg)] with a core sequence of cell adhesion molecules.

**DOI:** 10.1038/bjc.1989.40

**Published:** 1989-02

**Authors:** I. Saiki, J. Murata, J. Iida, N. Nishi, K. Sugimura, I. Azuma

**Affiliations:** Institute of Immunological Science, Hokkaido University, Sapporo, Japan.


*Br. J. Cancer* (1989), **59**, 194–197 © The Macmillan Press Ltd., 1989

SHORT COMMUNICATION

### The inhibition of murine lung metastasis by synthetic polypeptides [poly(arg-gly-asp) and poly(tyr-ile-gly-ser-arg)] with a core sequence of cell adhesion molecules

I. Saiki, J. Murata, J. Iida, N. Nishi[1], K. Sugimura & I. Azuma

*Institute of Immunological Science, Hokkaido University, Kita-15, Nishi-7, Kita-ku, Sapporo 060 and [1]Department of Polymer Science, Faculty of Science, Hokkaido University, Kita-10, Nishi-8, Kita-ku, Sapporo 060, Japan.*

During the sequential steps of metastases, tumour cells encounter various host cells (platelets, lymphocytes or endothelial cells) and/or extracellular matrix and basement membrane components (fibronectin or laminin) (Fidler, 1984). As a result of adhesive interaction, this encounter may lead to a multicellular embolus formation which can subsequently enhance the survival, arrest and invasiveness of tumour cells (Fidler, 1984; Terranova *et al.*, 1982, 1984). Since specific interactions between tumour cells and host cells or components are fundamental events in the metastatic process, the adhesion and detachment of the cells are therefore thought to be of prime importance for the control of the cellular functions of diverse cell types, including cells which are highly metastatic (Terranova *et al.*, 1984).

The molecules involved in the adhesion of both normal and tumour cells have been studied quite intensively in recent years. Fibronectin (Kornblihtt *et al.*, 1985), vitronectin (Suzuki *et al.*, 1985) and laminin (Sasaki *et al.*, 1987; Sasaki & Yamada, 1987) have been identified by molecular cloning as the primary structures of cell adhesion proteins. Common or characteristic core sequences responsible for cellular recognition in molecules have also been found to contribute to cell adhesion, spreading or migration (McCarthy & Furcht, 1984; Yamada & Kennedy, 1984; Rouslahti & Pierschbacher, 1987). The co-incubation of tumour cells with purified laminin followed by i.v. injection has been found to enhance pulmonary metastases, whereas a fragment of laminin inhibits the metastases (Barsky *et al.*, 1984). More recently, synthetic peptides containing core sequences (arg-gly-asp derived from fibronectin or tyr-ile-gly-ser-arg derived from laminin) have been shown to exhibit a similar inhibition of lung metastases (Humphries *et al.*, 1986; Iwamoto *et al.*, 1987). This evidence has prompted us to carry out an attempt to regulate more efficiently the mechanism involved in the adhesion of tumour cells during the metastatic process.

It is well known that the introduction of plural peptides (for example, peptide hormones) into carrier proteins can sometimes augment the activity of the peptide hormone because of the co-operative interaction between the molecules, although at the same time this may reduce molecular flexibility and mobility and consequently lead to a decrease in the affinity between the peptide and the specific receptors. Drastically high activity of the polymerised functional molecule has also been reported as a common phenomenon in the field of polymer catalyst or enzyme-model polymers, and has come to be called the 'polymer effect' (Kunitake & Okahata, 1976). We have found that poly-L-arg ($\sim$5,000 daltons in average molecular weight) is able to activate mouse peritoneal macrophages to become cytotoxic against tumour cells more effectively than $(\text{L-arg})_{12}$, $(\text{L-arg})_6$ and L-arg by i.p. administration (J. Iida *et al.*, manuscript submitted); this suggests that the polymerisation of L-arg plays a role in inducing the tumoricidal activity of macrophages. We therefore synthesised some polypeptides unique to our laboratory, poly(arg-gly-asp) or poly(tyr-ile-gly-ser-arg), which consist of repeated structures of the arg-gly-asp or tyr-ile-gly-ser-arg peptide sequences respectively, and poly(arg, gly, asp) which consists of the same amino acid components as poly(arg-gly-asp) but has a random sequence of amino acids. Polypeptides used in this study were prepared by the synthesis of the monomer peptides of arg-gly-asp or tyr-ile-gly-ser-arg sequences by the conventional method and a subsequent polymerisation reaction. *t*-Butoxycarbonyl (t-boc), mesitylenesulphonyl (mts) and methyl ($CH_3$) groups were employed as the protecting groups for α-amino guanidino and α-carboxyl groups. The benzyl (bzl) group was employed to protect the side-chain functional groups of asp, tyr and ser residues. The purity of the peptides were confirmed by thin layer chromatography and elemental analysis. Polymerisation of monomer peptide was carried out with diphenylphosphorylazide, as we have described elsewhere (Nishi *et al.*, 1980). The removal of the side-chain protecting groups from the resulting sequential polypeptides was carried out with a methansulphonic acid-anisole mixture for the initial polypeptide and with a trifluoromethanesulphonic acid-thioanisole-trifluoroacetic acid mixture for the latter polypeptide. The consequent methansulphonate or trifluoromethanesulphonate was converted to hydrochloride with Amberlite IRA 400 (Cl form) to give the final product. The complete removal of the protecting groups was confirmed by IR. The final products showed a typical polypeptide pattern. All the amino acids used in this study were of the L-form type. In the sequence of poly(arg-gly-asp), a gly residue is always left between the arg and asp residues, and the -arg-gly-asp- sequence exists as a block. In the sequence of poly(arg, gly, asp), on the other hand, these amino acids are randomly arranged without rule, and the probability of an -arg-gly-asp- sequence is statistically very small. Poly(arg-gly-asp) and its random polypeptide weigh $\sim$5,000 daltons while poly(tyr-ile-gly-ser-arg) weighs $\sim$10,000 daltons; this was assessed by viscometric measurements and SDS-polyacrylamide gel electrophoresis; they are then dissolved in phosphate-buffered saline (PBS) before use.

We first examined the adhesive capability of B16-BL6 melanoma cells to the polypeptide. $^{125}$I-iododeoxyuridine ($^{125}$I-IUdR) labelled B16-BL6 cells suspended in a serum-free MEM medium were added to microculture wells pre-coated with polypeptides or mouse fibronectin, and incubated at 37°C for 20 min. After they had been washed to remove unattached cells, the number of remaining substrate-bound tumour cells was calculated by measuring their radioactivity (Saiki *et al.*, 1986). Poly(arg-gly-asp) and fibronectin promoted the adhesion of B16-BL6 cells (Table I). However, few B16-BL6 cells attached themselves to the substrates coated with poly(arg, gly, asp) or to bovine serum albumin (BSA) used as a negative control. To investigate the

Correspondence: I. Saiki.
Received 1 July 1988, and in revised form, 6 October 1988.

**Table I** Adhesion of B16-BL6 melanoma cells to polypeptide- or fibronetin-coated substrates

| *Coated with* | *Co-incubated with* | | *Binding capacity No. of cells bound/substrate* ± *s.d.*[a] |
|---|---|---|---|
| Fibronectin | | | 5,849 ± 513 |
| Poly(arg-gly-asp) | | | 7,967 ± 910 |
| Poly(arg, gly, asp) | | | 1,750 ± 395 |
| BSA | | | 1,239 ± 347 |
| Fibronectin | + arg-gly-asp | 500 μg ml$^{-1}$ | 3,708 ± 265 (37%) |
| | | 100 | 5,320 ± 52 |
| | + his-gly-gly | 500 | 6,133 ± 787 |
| | + poly(arg-gly-asp) | 500 | 3,715 ± 231 (36%) |
| | | 100 | 3,687 ± 229 (37%) |

$^{125}$I-IUdR labelled B16-BL6 cells ($2 \times 10^4$) suspended in serum-free medium were added to wells coated with 5 μg ml$^{-1}$ fibronectin, 20 μg ml$^{-1}$ polypeptides or 1% BSA in PBS, in the absence or presence of peptides. After 20 min incubation, non-adherent cells were washed away and the remaining adhered cells were counted. [a]Mean ± s.d. in triplicate cultures. The results of a representative sample of four independent experiments are shown.

specificity of cell adhesion to fibronectin-coated substrate, we carried out a cell adhesion assay in the presence of tripeptides or polypeptides. Arg-gly-asp and poly(arg-gly-asp) were specifically able to inhibit the adhesion of tumour cells to fibronectin-coated substrates, whereas the unrelated sequencing tripeptide his-gly-gly were not able to do so. The inhibition was caused by co-incubation with increasing amounts of the arg-gly-asp sequence in a dose-dependent manner or by the addition of 5 mM EDTA (I. Saiki *et al.*, manuscript submitted). Poly(arg-gly-asp) showed a clear ability to inhibit the cell adhesion to fibronectin several times on a dose (weight), and did so more effectively than arg-gly-asp tripeptide. These results indicate that the cell-adhesion promoting activity of poly(arg-gly-asp) as well as fibronectin depends on the specific mechanism mediated by the arg-gly-asp sequence in a cation-dependent manner; this suggests that the cell surface receptor responsible for adhesion is able to recognise this sequence in the adhesion molecule (Cheresh *et al.*, 1987).

We next considered whether our synthetic polypeptides are able or not to inhibit lung metastases caused by the i.v. injection of tumour cells. To do this, we used two highly metastatic tumour cells, B16-BL6 melanoma and Lewis lung carcinoma (3LL). Co-injection of 500 μg poly(arg-gly-asp) with $5 \times 10^4$ B16-BL6 cells or $3 \times 10^5$ 3LL cells caused a significant reduction of lung metastases in C57BL/6 mice ($P < 0.001$ respectively), but 500 μg of either poly(arg, gly, asp) or arg-gly-asp tripeptide did not (Table II). Nevertheless, a significant inhibition of lung metastases was observed when the dose (3,000 μg mouse$^{-1}$) of arg-gly-asp tripeptide was increased ($P < 0.001$). The inhibition caused by tripeptide is similar to one reported previously, that substantial inhibition of tumour metastases of B16-F10 cells can be obtained with 3 mg of pentapeptide containing arg-gly-asp (Humphries *et al.*, 1986). Poly(tyr-ile-gly-ser-arg) at all doses used in this study inhibited significantly the lung metastases, but tyr-ile-gly-ser-arg pentapeptide did not inhibit the metastases at any dose except one of 200 μg (Table II). Similar inhibitory effects were obtained in the experimental metastasis model using 3LL. These results thus clearly demonstrate that poly(arg-gly-asp) or poly(tyr-ile-gly-ser-arg) could inhibit the lung metastases ~5–10 times more efficiently than the arg-gly-asp or tyr-ile-gly-ser-arg peptides. In addition, the i.v. injection of poly(arg-gly-asp) following an injection of B16-BL6 cells (i.e. sequential separate injection) was almost as effective a means of reducing the tumour colonies in the lung as the co-injection (premixing) of cells and poly(arg-gly-asp) (I. Saiki *et al.*, manuscript submitted). The polypeptides used in this study had no harmful cytotoxic effects on such cells as the B16-BL6 cells, 3LL cells, mouse red blood cells or thymocytes, nor did it affect their cell growth or the aggregation of the serum proteins. We have also observed that the inhibition of lung metastases can be induced by co-injection with various soluble polypeptide analogues containing the arg-gly-asp sequence, and even with a polypeptide entrapped (insolubilised) within non-phagocytisable multilamellar liposome membranes (I. Saiki *et al.*, manuscript submitted). These findings may imply that poly(arg-gly-asp) or poly(tyr-ile-gly-ser-arg) have higher affinity for adhesion receptors or have molecule conformations or environments of polypeptide more appropriate to the adhesion receptors than arg-gly-asp or tyr-ile-gly-ser-arg peptides. These points need to be studied further.

In our next experiment, we examined whether or not the lung metastases of B16-BL6 melanoma could be inhibited by the intralesional administration of polypeptides into the established primary tumour in the spontaneous metastasis model. Poly(arg-gly-asp) was administered intratumorally (or intralesionally) into the right hind footpad with an advanced primary tumour at various times following tumour inoculation, after which, on day 21, the primary tumours were surgically removed. Tumour colonies in the lung were monitored 14 days after tumour excision. The results of a representative sample of the three independent experiments are shown in Table III. Single or multiple intratumoral administrations of poly(arg-gly-asp) on day 1, day 7 or day 7, 10, 13, 16 caused a marked reduction of tumour colonies of B16-BL6 melanoma, but did not affect the growth (size) of primary tumours on day 21 compared with untreated control. The administration of random polypeptide, poly(arg, gly, asp), on day 7 after tumour inoculation was not able to inhibit the lung metastases. These results indicate that the inhibition of lung metastases by means of the intratumoral administration of poly(arg-gly-asp) may depend on the inhibition of active migration of tumour cells away from the primary tumour site. Furthermore, we also observed that multiple systemic administration of poly(arg-gly-asp) on days 7, 9, 11, 13, 15, 17 and 19 after an intrafootpad inoculation of the tumour led to a significant decrease of the lung tumour colonisation in the spontaneous model (I. Saiki *et al.*, manuscript submitted).

The exact mechanism responsible for the inhibition of lung metastases by these polypeptides may thus be more complex than a simple blockage of cell adhesion. We have recently observed that poly(arg-gly-asp) inhibits tumour-induced platelet aggregation which in turn is responsible for the enhancement of tumour cell arrest in the capillaries (Gasic *et al.*, 1973; Jamieson *et al.*, 1987), but does not directly provoke the aggregation of platelets (Saiki *et al.*, 1988). Poly(tyr-ile-gly-ser-arg) is specifically able to inhibit the penetration of melanoma cells to the membrane filters precoated with laminin on the lower surface (haptotactic migration) in a dose-dependent manner (Murata *et al.*, 1988). Some possibilities also include the acceleration of release of arrested tumour cells from the lung and the inhibition by polypeptide of their lodgement.

In conclusion, we demonstrated that unique polypeptides

**Table II** Effect of polypeptides on experimental lung metastases induced by injection with metastatic tumour cells

| *Tumours* | *Administered i.v. with*[a] | *Dose* ($\mu g$ *mouse*$^{-1}$) | *No. of lung metastases Mean* ± *s.d.* (*range*) | $P^b$ |
|---|---|---|---|---|
| *B16-BL6* | | | | |
| Expt 1 | Untreated (PBS) | – | 91±19 (64–112) | |
| | Poly(arg-gly-asp) | 500 | 23± 3 (20– 28) | <0.001 |
| | Arg-gly-asp | 3,000 | 18±24 (1– 52) | <0.001 |
| | | 500 | 69±21 (50–102) | |
| | Poly(arg, gly, asp) | 500 | 65± 4 (64– 68) | |
| Expt 2 | Untreated (PBS) | – | 115±24 (86–149) | |
| | Poly(tyr-ile-gly-ser-arg) | 200 | 0 (0) | <0.001 |
| | | 100 | 19± 9 (10– 32) | <0.001 |
| | | 20 | 43±13 (24– 56) | <0.001 |
| | | 5 | 81± 9 (67– 89) | <0.02 |
| | Tyr-ile-gly-ser-arg | 200 | 35±16 (18– 60) | <0.001 |
| | | 100 | 101±17 (77–122) | |
| | | 20 | 109±39 (61–164) | |
| | | 5 | 128±31 (104–180) | |
| 3LL | Untreated (PBS) | – | 139±19 (113–158) | |
| | Poly(arg-gly-asp) | 500 | 34± 7 (26– 41) | <0.001 |
| | Poly(tyr-ile-gly-ser-arg) | 100 | 14±11 (2– 28) | <0.001 |
| | Tyr-ile-gly-ser-arg | 100 | 123±45 (74–187) | |

[a]B16-BL6 cells ($5 \times 10^4$ per 0.2 ml) or 3LL ($3 \times 10^5$ per 0.2 ml) were injected i.v. with or without admixing with polypeptides into five mice per group. Lung tumour colonies were examined 14 days later. The results of a representative sample of several independent experiments are shown. [b]Compared with untreated control (PBS) by Student's two-tailed *t* test.

**Table III** Therapeutic effect of polypeptides on spontaneous lung metastases by intrafootpad administration of B16-BL6 melanoma cells

| *Treated with* | *Dose* ($\mu g$ *mouse*$^{-1}$) | *Timing* | *Primary tumour size on day 21* (*mm* ± *s.d.*) | *No. of lung metastases Mean* ± *s.d.* (*range*) | $P^a$ |
|---|---|---|---|---|---|
| Untreated (PBS) | | | 10±4 | 129±38 (78–180) | |
| Poly(arg-gly-asp) | 100 | on day 1 | 8±4 | 24±17 (4– 49) | <0.001 |
| | | on day 7 | 9±3 | 29±22 (0– 50) | <0.001 |
| | | on day 14 | 10±4 | 120±29 (79–155) | |
| | | on day 20 | 8±3 | 131±79 (75–247) | |
| | 50×4 | on day 7, 10, 13, 16 | 8±3 | 26±29 (0– 68) | <0.01 |
| Poly(arg, gly, asp) | 100 | on day 7 | 11±3 | 158±62 (117–230) | |

Five C57BL/6 mice per group were administered intratumorally with 100 or 50 $\mu g$ of polypeptides (0.05 ml) at the indicated times after the intrafootpad injection ($5 \times 10^5$ per 0.05 ml) of B16-BL6 melanoma cells. Primary tumours were surgically removed on day 21 and mice were killed two weeks after tumour excision. The results of a representative of three experiments are shown. [a]Compared with the untreated group by Student's two-tailed *t* test.

containing the repetitive structure of arg-gly-asp or tyr-ile-gly-ser-arg core sequences are able to inhibit tumour lung metastases in experimental and spontaneous metastases models, possibly by means of their ability to interfere with the cellular adhesive process of metastases, and that multivalent units of the arg-gly-asp or tyr-ile-gly-ser-arg core sequences are able to promote the inhibition of the lung metastases more dramatically than single units: this evidence indicates the prominent effect of sequential polymerisation. The mechanism for the inhibition of lung metastases by these polypeptides is now being examined in detail. A core sequence containing polypeptides taken from cell adhesion molecules may thus provide a promising basis for the prevention of cancer metastases.

This work was supported in part by grants-in-aid for cancer research from the Japanese Ministry of Education, Science and Culture; from the Japanese Ministry of Health and Welfare for comprehensive 10-year strategy for cancer control; for scientific research and for developmental scientific research (No. 62870023) from the Japanese Ministry of Education, Science and Culture; for scientific research from the Japanese Ministry of Education, Science and Culture; by the Osaka Foundation for Promotion of Clinical Immunology; by Yamaouchi Foundation for Research on Metabolic Disorders; and grant-in-aid for special project research from Hokkaido University, Japan. The authors thank Ms M. Araki for typing the manuscript.

### References

BARSKY, S.H., RAO, C.N., WILLIAMS, J.E. & LIOTTA, L.A. (1984). Laminin molecular domains which alter metastasis in a murine model. *J. Clin. Invest.*, **74,** 843.

CHERESH, D.A., PYTELA, R., PIERSCHBACHER, M.D., KLIER, F.G., RUOSLAHTI, E. & REISFELD, R.A. (1987). An arg-gly-asp-directed receptor on the surface of human melanoma cells exist in a divalent cation-dependent functional complex with the disialoganglioside GD2. *J. Cell Biol.*, **105,** 1163.

FIDLER, I.J. (1984). The Ernst W. Bertner Memorial Award Lecture: The evolution of biological heterogeneity in metastatic neoplasms. In *Cancer Invasion and Metastases: Biologic and Therapeutic Aspects*, Nicolson, G.L. & Milas, L. (eds) p. 5. Raven Press: New York.

GASIC, G.J., GASIC, T.B., GALANTI, N., JOHNSON, T. & MURPHY, S. (1973). Platelet tumor interactions in mice. The role of platelets in the spread of malignant disease. *Int. J. Cancer*, **11,** 704.

HUMPHRIES, M.J., OLDEN, K. & YAMADA, K.M. (1986). A synthetic peptide from fibronectin inhibits experimental metastasis of murine melanoma cells. *Science*, **233**, 467.

IWAMOTO, Y., ROBEY, F.A., GRAF, J. & 4 others (1987). YIGSR, a synthetic laminin pentapeptide, inhibits experimental metastasis formation. *Science*, **238**, 1132.

JAMIESON, G.A., BASTIDA, E. & ORDINAS, A. (1987). Interaction of platelets and tumour cells. In *Platelets in Biology and Pathology III*, MacIntryere, D.E. & Gordon, J.L. (eds) p. 161. Elsevier Science Publishers: New York.

KORNBLIHTT, A.R., UMEZAKA, K., VIBE-PEDERSAN, K. & BARALLE, F.E. (1985). Primary structure of human fibronectin: differential splicing may generate at least 10 polypeptides from a single gene. *EMBO J.*, **4**, 1755.

KUNITAKE, T. & OKAHATA, Y. (1976). Catalytic hydrolysis by synthetic polymers. *Adv. Polymer. Sci.*, **20**, 159.

McCARTHY, J.B. & FURCHT, L.T. (1984). Laminin and fibronectin promote the haptotactic migration of B16 mouse melanoma cells *in vitro*. *J. Cell Biol.*, **98**, 1474.

MURATA, J., SAIKI, I., NISHI, N. & AZUMA, I. (1988). Inhibitory effect of a synthetic polypeptide, poly (Tyr-Ile-Gly-Ser-Arg), on the metastatic formation of malignant tumor cells. *Int. J. Biol. Macromol.* (in press).

NISHI, N., NAKAJIMA, B., HASEBE, N. & NOGUCHI, J. (1980). Polymerization of amino acid or peptides with diphenylphosophoryl azide (DPPA). *Int. J. Biol. Macromol.*, **2**, 53.

ROUSLAHTI, E. & PIERSCHBACHER, M.D. (1987). New perspectives in cell adhesion: RGD and integrins. *Science*, **238**, 4910.

SAIKI, I., NAYAR, R., BUCANA, C. & FIDLER, I.J. (1986). A microassay for the rapid and selective binding of cells from solid tumors to mouse macrophages. *Cancer Immunol. Immunother.*, **22**, 125.

SAIKI, I., NISHI, N., IIDA, J., MATSUNO, K. & AZUMA, I. (1988). Biological activities of synthetic polypeptide containing a repetitive core sequence (Arg-Gly-Asp) of cell adhesion molecules. *Int. J. Biol. Macromol.*, **10**, in press.

SASAKI, M., KATO, S., KOHNO, K., MARTIN, G.R. & YAMADA, Y. (1987). Sequence of the cDNA encoding the laminin B1 chain reveals a multi-domain protein containing cystein rich repeats. *Proc. Natl Acad. Sci. USA*, **84**, 935.

SASAKI, M. & YAMADA, Y. (1987). The laminin B2 chain has a multidomain structure homologous to the B1 chain. *J. Biol. Chem.*, **262**, 17111.

SUZUKI, S., OLDBERG, A., HAYMAN, E.G., PIERSCHBACHER, M.D. & ROUSLAHTI, E. (1985). Complete amino acid sequence of human vitronectin deduced from cDNA. Similarity of cell attachment sites in vitronectin and fibronectin. *EMBO J.*, **4**, 2519.

TERRANOVA, V.P., LIOTTA, L.A., RUSSO, R.G. & MARTIN, G.R. (1982). Role of laminin in the attachment and metastasis of murine tumor cells. *Cancer Res.*, **42**, 2265.

TERRANOVA, V.P., WILLIAMS, J.E., LIOTTA, L.A. & MARTIN, G.R. (1984). Modulation of the metastatic activity of melanoma cells by laminin and fibronectin. *Science*, **226**, 982.

YAMADA, K.M. & KENNEDY, D.W. (1984). Dualistic nature of adhesive protein function: fibronectin and its biologically active peptide fragments can autoinhibit fibronectin function. *J. Cell Biol.*, **99**, 29.